# Development and validation of a predictive model for the progression of diabetic kidney disease to kidney failure

**DOI:** 10.1080/0886022X.2020.1772294

**Published:** 2020-06-11

**Authors:** Yaqi Cheng, Jin Shang, Dong Liu, Jing Xiao, Zhanzheng Zhao

**Affiliations:** Department of Nephrology, The First Affiliated Hospital of Zhengzhou University, Zhengzhou, China

**Keywords:** Diabetic kidney disease, renal replacement, predictive model, progression

## Abstract

**Introduction:**

A good prediction model plays an important role in determining the progression to diabetic kidney disease. We aimed to create a model to predict progression to kidney failure in patients with diabetic kidney disease.

**Methods:**

We retrospectively assessed 641 patients with type 2 diabetic kidney disease as derivation cohort and 280 patients as external out time validation cohort. We used a combination of clinical guidance and univariate logistic regression to select the relevant variables. We calculated the discrimination and calibration of different models. The best model was selected according to the optimal combination of discrimination and calibration.

**Results:**

During the 3 years follow up, there were 272 outcomes (42%) in derivation cohort and 138 outcomes (49%) in external validation cohort. The final variables selected in the multivariate logistics regression were age, gender, hemoglobin, NLR, serum cystatin C, eGFR, 24-h urine protein, and the use of oral hypoglycemic drugs. We developed four different models as clinical, laboratory, lab-medication, and full models according to these independent risk factors. Laboratory model performed well in both discrimination and calibration among all the models (C-statistics: external validation 0.863; *p* value of the Hosmer–Lemeshow, .817). There was no significant difference in NRI among laboratory model, lab-medication model, and full model (*p* > .05). So, we chose the laboratory model as the optimal model.

**Conclusion:**

We constructed a nomogram which contained hemoglobin, NLR, serum cystatin C, eGFR, and 24-h urine protein to predict the risk of patients with diabetic kidney disease initiating renal replacement in 3 years.

## 1. Introduction

Despite advances over the past 20 years in delaying the progression of diabetic kidney disease (DKD), it is still a leading cause of end-stage renal disease (ESRD) worldwide, accounting for approximately 50% of cases in the developed world [1], and it imposes a heavy burden not only on individual patients but also on society [2]. The costs associated with ESRD in the United States reached $34.3 billion, accounting for 6.3% of the Medicare budget in 2013 [3]. The overall costs of care for patients with DKD are extraordinarily high. For example, overall Medicare expenditures for diabetes and chronic kidney disease (CKD) in the mostly older (more than 65 years of age) medicare population were approximately $25 billion in 2011 [1]. As a cross-sectional survey in 2012 demonstrated that the overall prevalence of chronic kidney disease was 10.8% (10.2–11.3) in China; therefore, the number of patients with chronic kidney disease in China is estimated to be about 119.5 million (112.9–125.0 million) [4]. Diabetes is one of the major non-communicable diseases that cause the kidney damage. Therefore, it is important to be able to predict the risk of initiating renal replacement in patients with DKD so that we could intervene earlier and allocate medical resources better to high-risk patients.

There are several prediction models that predict the progression of chronic kidney disease or diabetic kidney disease. Tangri et al. developed the kidney failure risk equation (KFRE) to identify a high-risk population of CKD 3-5 patients who are likely to developing ESRD based on demographic, clinical, and laboratory variables [5]. The simple version of the KFRE uses only four clinical variables (age, sex, the estimated glomerular filtration rate [eGFR], and the urine albumin/creatinine ratio [ACR]) to identify patients who are at high risk of developing ESRD with a C-statistic >0.90 (95% CI, 0.894–0.926; *p* < .001); this equation has been verified in 721,357 participants with CKD stages 3 to 5 in more than 30 countries across four continents with very similar C-statistic values [6]. However, the KFRE does not account for the etiology of CKD, so it plays an important role in decision-making for patients with CKD other than DKD. Due to the clinical manifestations of DKD, patients with DKD have a much higher risk of progressing to ESRD than other CKD patients [1,7–9]. It has been unclear whether the KFRE can detect DKD patients at high risk patients of progressing to ESRD. Wan et al. developed new gender-specific models provide a more accurate 5-year ESRD risk predictions for Chinese diabetic primary care patients than other existing models such as the ADVANCE and New Zealand models [10]. But the aim of this study was to develop a 5-year ESRD risk prediction model among Chinese patients with Type 2 diabetes mellitus instead of DKD. Moreover the developed models in this study contained 11–12 predictors, which may be difficult to be applied in clinical practices. Dunkler et al. used the Ramipril Global Endpoint Trial (ONTARGET) and Outcome Reduction with Initial Glargine Intervention (ORIGIN) cohort to develop two risk prediction models for early CKD in Type 2 Diabetes [11]. However, the participants of these two cohorts included type 2 diabetes with normoalbuminuria or microalbuminuria at baseline other than just patients with DKD. What’s more, the outcome of this study was incidence or progression of CKD, which was defined as new microalbuminuria or macroalbuminuria, doubling of creatinine, or ESRD.

Therefore, we aimed to develop and validate a model to predict the 3-year risk of progression to kidney failure that could be easily implemented in clinical practice, using the variables routinely measured in patients with DKD.

## 2. Materials and methods

### 2.1. Study population and design

In this population-based retrospective cohort study, patients with type 2 DKD who were hospitalized in the First Hospital Affiliated with Zhengzhou University were screened. A total of 641 patients who were hospitalized from December 2012 to December 2015 were enrolled as derivation cohort, while 280 patients hospitalized from January 2016 to December 2017 were enrolled as external out time validation cohort. The DKD diagnosis was based on the guidelines created by the Kidney Disease: Improving Global Outcomes [12]. The inclusion criteria were as follows: (i) a verified diagnosis of type 2 diabetes; and (ii) persistent albuminuria or decreased renal function; or (iii) renal biopsy-certified kidney disease caused by diabetes. The exclusion criteria were as follows: (i) patients who did not complete 3 years of follow-up; (ii) a pathological diagnosis of DKD combined with nondiabetic nephropathy, such as membranous nephropathy or IgA nephropathy; (iii) patients who had systemic diseases, such as allergic purpura, vasculitis, and systemic lupus erythematosus, which might cause persistent albuminuria or decreased renal function; and (iv) patients who underwent renal replacement prior to admission.

### 2.2. Data collection

Clinical characteristics, including age, sex, duration of diabetes mellitus (DM), systolic blood pressure, diastolic blood pressure, the main medical history of hypertension, coronary heart disease and cancer, body mass index (BMI) and the use as the drugs such as the oral hypoglycemic drugs, insulin, renin-angiotensin system blocker, and statin were collected. Laboratory parameters, including hemoglobin, neutrophil: lymphocyte ratio (NLR), serum creatinine level, serum urea level, serum uric acid level, serum total protein level, serum albumin level, serum calcium level, total cholesterol level, triglyceride level, serum cystatin C level, serum β 2 microglobulin level, glycated hemoglobin (HbA1c) level, blood glucose level, C-reactive protein (CRP) level, erythrocyte sedimentation rate (ESR), estimated glomerular filtration rate (eGFR, calculated by the CKD-EPI formula), 24-h urine protein level, bicarbonate level and the PH value of urine were initially collected at the time of diagnosis with DKD.

### 2.3. Incident outcome

The primary endpoint was kidney failure, as defined by the initiation of dialysis and renal transplantation.

### 2.4. Statistical analysis

There were some missing data in several variables. Variables that indicated the renal function, such as serum creatine, serum urea, serum cystatin C, serum β2 microglobulin, and serum uric acid, had 3.9% missing values. There were 2.9% missing data in hemoglobin and NLR and 6.1% in serum total protein and serum albumin. The 24-h urine protein had 3.1% missing values and CRP and ESR had 8.4% missing data. We used multivariate multiple imputation with chained equations to impute missing values to maximize the statistical power and diminish bias [13]. First, we use the means ± standard deviation (*SD*) or medians (quartile 1, quartile 3) to express the continuous data, while the categorical data are expressed as the absolute values and percentages. Then, we used the univariate logistic regression to select the potential variables. Before the multiple logistic regression, we calculated the collinearity of the variables and remove the factors, serum urea level and serum creatine level, that exist in collinearity. Then, we used a combination of clinical guidance and univariate logistic regression with the enter method to select the relevant variables and conduct multiple logistic regression. The clinical characteristics, including age, sex and the use of oral hypoglycemic drugs, and the statistically significant biochemical measures, including the hemoglobin level, NLR, serum cystatin C level, eGFR, and 24-h urine protein level, were selected to construct the sequential models. Next, the area under the receiver operating characteristic curve, which is referred to as the C-statistic, was used to assess the discriminatory ability of the models. The calibration was assessed by the Hosmer–Lemeshow (H-L) test and calibration curves. The goodness of fit of the models was evaluated by the Akaike information criterion (AIC). We divided all the derivation patients into five groups and selected one group as the internal validation cohort and the other four groups as the training cohort. We used the training cohort to develop the models and the internal validation cohort to validate the models. We repeated the process 5 times to assess the discriminatory ability and calibration of the models. The C-statistic, the AIC, and the *p* value of the Hosmer–Lemeshow (H-L) test shown were the averages of 5 repetitions, and the detailed values for each individuals repetition are displayed in the Supplementary Materials. The best model was selected according to the optimal combination of the discrimination and calibration calculated by the fivefold crossvalidation and external validation which is ‘Bootstrapping’. The net reclassification improvement and integrated discrimination improvement of models were also used to evaluate the discriminatory ability of the models. Last, we constructed a nomogram to predict the risk of a patient with DKD initiating renal replacement in 3 years. All analyses involved in the development and validation of the model were conducted with R software, version 3.5.3 (R Project for Statistical Computing).

## 3. Results

### 3.1. Cohort description

The baseline clinical characteristics and laboratory parameters of the derivation cohort and external validation cohort are shown in Table 1 and Table 2. During the 3 years follow up, there were 272 outcomes (42%) in derivation cohort and 138 outcomes (49%) in external validation cohort. There were no significant differences in medical history of coronary heart disease and cancer, BMI, the use of oral hypoglycemic drugs, NLR, serum creatine, serum urea, serum cystatin C, serum urine acid, serum β2 hemoglobulin, eGFR, blood glucose, glycated hemoglobin, bicarbonate, and PH value of urine between the derivation cohort and external validation cohort. Compared to the patients in derivation cohort, the patients in external validation cohort had lower duration of DM, hemoglobin levels, serum total protein levels, serum albumin levels, C-reactive protein levels, serum calcium levels and higher blood pressure, and 24-h urine protein levels. Moreover, the use of insulin, RASB and statin were significantly different between the two cohort.

**Table 1.** Baseline clinical characteristics of the study participants.

Characteristics	Derivation cohort (*n* = 641)	External validation (*n* = 280)	*p* Value
Age, years	56 (48, 64)	51 (45, 59)	<.001
Male sex (%)	381 (59)	113 (40)	<.001
Duration of DM, years	10 (5, 16)	8.5 (3.75, 13)	<.001
Main Medical History			
Hypertension (%)	449 (70)	223 (80)	.003
Coronary heart disease (%)	58 (9)	33 (10)	.408
Cancer (%)	6 (1)	2 (1)	.686
Blood pressure			
Systolic blood pressure (mmHg)	142 (130, 160)	146 (135, 165)	.002
Diastolic blood pressure (mmHg)	84 (77, 90)	88 (80, 96)	<.001
BMI (kg/m^2^)	25.35 (23.05, 28.04)	25.34 (22.97, 27.92)	.783
Oral hypoglycemic drugs			.199
No (%)	399 (62)	161 (57)	
Yes (%)	242 (38)	119 (42)	
Insulin			<.001
No (%)	226 (35)	173 (62)	
Yes (%)	415 (65)	107 (38)	
RASB			<.001
No (%)	427 (67)	71 (25)	
Yes (%)	214 (33)	209 (75)	
Statin			.001
No (%)	383 (60)	134 (48)	
Yes (%)	258 (40)	146 (52)	
Outcome			.064
No (%)	369 (58)	142 (51)	
Yes (%)	272 (42)	138 (49)	

DM: diabetes mellitus; BMI: body mass index; RASB: renin-angiotensin system blocker.

### 3.2. Predictive risk factors

The odds ratios and *p* value of univariate logistic regression were shown in Table 3, which can be used to select the potential risk factors. Patients who had higher serum cystatin C levels (OR, 7.481; 95% CI, 5.570–10.498; *p* < .001), higher β2 microglobulin levels (OR, 1.913; 95% CI, 1.750–2.111; *p* < .001), higher 24-h urine protein levels (OR, 2.081; 95% CI, 1.858–2.362 *p* < .001) and higher NLRs (OR, 1.514; 95% CI, 1.374–1.686; *p* < .001) had a higher risk of undergoing renal replacement. In addition, patients who had higher serum calcium levels (OR, 0.0005; 95% CI, 0.0001–0.002; *p* < .001) and higher serum albumin levels (OR, 0.870; 95% CI, 0.848–0.893; *p* < .001) had a lower risk of renal replacement. There are also other indicators that are statistically significant in univariate analysis (*p* < .05). We conducted the collinear analysis in Table 4 before multivariate analysis to eliminate the effect of collinearity. Consequently, serum creatine and serum urea were taken out in multivariate analysis due to VIF was more than 5, which indicates that they have collinearity with eGFR (Table 4). The final variables selected in the multivariate logistics regression were age (OR, 0.949; 95% CI, 0.913–0.985; *p*, .007), gender (OR, 2.952; 95% CI, 1.196–7.718; *p*, 0.022), hemoglobin (OR, 0.970; 95% CI, 0.948–0.990; *p*, 0.004), NLR (OR, 1.099; 95% CI, 1.008–1.198; *p*, .028), serum cystatin C (OR, 1.932; 95% CI, 1.369–2.804; *p* < .001), eGFR (OR, 0.945; 95% CI, 0.924–0.964; *p* < .001), 24-h urine protein (OR, 1.215; 95% CI, 1.055–1.427; *p*, .011) and the use of oral hypoglycemic drugs (OR, 0.373; 95% CI, 0.143–0.941; *p*, .039; Table 5).

**Table 3.** Potential risk factors identified by univariate logistic regression analysis.

	OR	95% CI		*p* Value
Age	1.005	0.992	1.018	.475
Gender	1.152	0.837	1.588	.380
BMI	0.990	0.957	1.023	.535
Duration of DM	1.047	1.024	1.071	<.001
Hypertension	3.099	2.138	4.555	<.001
Coronary heart disease	1.297	0.752	2.229	.346
Cancer	1.361	0.250	7.401	.707
Systolic blood pressure	1.031	1.022	1.040	<.001
Diastolic blood pressure	1.014	1.000	1.028	.050
Hemoglobin	0.917	0.905	0.928	<.001
NLR	1.514	1.374	1.686	<.001
Serum creatinine	1.015	1.012	1.017	<.001
Serum urea	1.406	1.340	1.485	<.001
Serum uric acid	1.010	1.008	1.012	<.001
Serum total protein	0.888	0.866	0.909	<.001
Serum albumin	0.870	0.848	0.893	<.001
Total cholesterol	1.199	1.068	1.350	.002
Triglyceride	0.966	0.871	1.064	.486
Serum calcium	0.0005	0.0001	0.002	<.001
Serum cystatin C	7.481	5.570	10.498	<.001
β 2 microglobulin	1.913	1.750	2.111	<.001
Glycated hemoglobin	0.624	0.560	0.691	<.001
C-reactive protein	1.013	1.006	1.022	.001
ESR	1.038	1.031	1.045	<.001
24-h urine protein	2.081	1.858	2.362	<.001
eGFR	0.911	0.895	0.925	<.001
Blood glucose	0.976	0.930	1.025	.335
Bicarbonate	0.926	0.891	0.961	<.001
PH value of urine	1.137	0.940	1.376	.185
Oral hypoglycemic drugs use	0.300	0.210	0.424	<.001
Insulin use	0.621	0.447	0.861	.004
RASB use	0.593	0.421	0.832	.002
Statin use	0.697	0.503	0.961	.028

DM: diabetes mellitus; BMI: body mass index; NLR: neutrophil: lymphocyte ratio; ESR: erythrocyte sedimentation rate; eGFR: estimated glomerular filtration rate; RASB: renin-angiotensin system blocker.

**Table 4.** Collinear analysis of potential risk factors.

	Tolerance	VIF
Age	0.719	1.390
Gender	0.819	1.222
Duration of DM	0.763	1.310
hypertension	0.751	1.331
Systolic blood pressure	0.646	1.547
Diastolic blood pressure	0.777	1.286
Hemoglobin	0.287	3.481
NLR	0.385	2.599
Serum creatinine	0.136	7.372
Serum urea	0.181	5.511
Serum uric acid	0.481	2.080
Serum total protein	0.518	1.932
Serum albumin	0.554	1.805
Total cholesterol	0.741	1.349
Serum calcium	0.946	1.057
Serum cystatin C	0.226	4.421
β 2 microglobulin	0.284	3.516
Glycated hemoglobin	0.790	1.265
C-reactive protein	0.500	2.001
ESR	0.533	1.874
24-h urine protein	0.510	1.960
eGFR	0.124	8.041
Bicarbonate	0.878	1.139
Oral hypoglycemic drugs use	0.796	1.257
Insulin use	0.906	1.104
RASB use	0.907	1.103
Statin use	0.895	1.117

DM: diabetes mellitus; NLR: neutrophil: lymphocyte ratio; ESR: erythrocyte sedimentation rate; eGFR: estimated glomerular filtration rate; RASB: renin-angiotensin system blocker.

**Table 5.** Potential risk factors identified by multivariate logistic regression analysis.

	OR	95% CI		*p* Value
Age	0.949	0.913	0.985	.007
Gender	2.952	1.196	7.718	.022
Hemoglobin	0.970	0.948	0.990	.004
NLR	1.099	1.008	1.198	.028
Serum cystatin C	1.932	1.369	2.804	<.001
24-h urine protein	1.215	1.055	1.427	.011
eGFR	0.945	0.924	0.964	<.001
Oral hypoglycemic drugs use	0.373	0.143	0.941	.039

NLR: neutrophil: lymphocyte ratio; eGFR: estimated glomerular filtration rate.

### 3.3. Prediction model performance in the cohort

**Figure 1.**
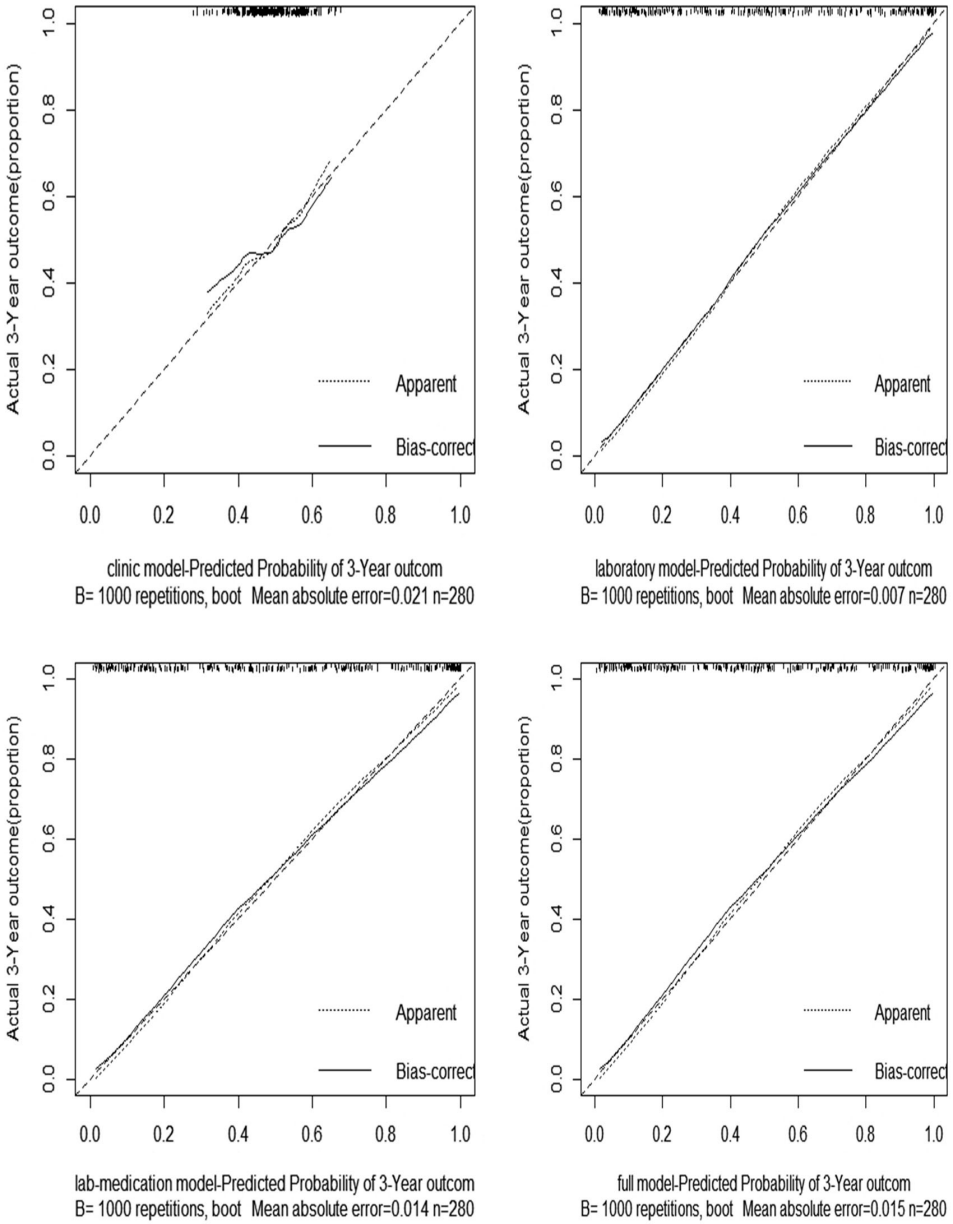
Calibration curves of four models.

We constructed sequential models using the variables selected by multivariate logistic regression analysis in Table 5. The performance for the clinical model, laboratory model, lab-medication model, and full model were reported in Table 6. The calibration curves calculated by external validation of four models were shown in Figure 1. As we have mentioned before, the C-statistic, AIC, *p* value of the Hosmer–Lemeshow (H-L) test, and calibration curves were used to depict the discriminatory ability and calibration of the models. A higher C-statistic reflected a good discriminatory ability, while a *p* value of the Hosmer–Lemeshow (H-L) test close to 1 and a relatively lower AIC suggested that the predicted probability was not significantly different from the observed probability in the models. Clinical model, including age, gender and the use of oral hypoglycemic drugs only, performed poorly in both the training cohort (C-statistic, 0.626; *p* value of H-L test, 0.315) and the validation cohort (internal: C-statistic, 0.616; *p* value of H-L test, 0.524; external: C-statistic, 0.506). The remaining three models, laboratory model, lab-medication model, and full model, had similar C statistics in both internal and external validation (Table 6). However, the full model, which included age, gender, hemoglobin, NLR, serum cystatin C, eGFR, 24-h urine protein, and the use of the oral hypoglycemic drugs showed the poor *p* value of the Hosmer–Lemeshow test with 0.510 in internal validation. The lab-medication model, which included hemoglobin, NLR, serum cystatin C, eGFR, 24-h urine protein, and the use of the oral hypoglycemic drugs showed the poor *p* value of the Hosmer–Lemeshow test with 0.438 in training cohort. Thus, the laboratory model, including hemoglobin, NLR, serum cystatin C, eGFR, and 24-h urine protein, had good discriminatory ability and good calibration.

**Table 6.** Performances of the sequential models with different combinations of predictive variables in the derivation and validation cohorts.

	Clinical model	Laboratory model	Lab-medication model	Full model
Age	1.005			0.950
Gender	1.174			2.560
Hemoglobin		0.973	0.975	0.969
NLR		1.105	1.094	1.098
Serum cystatin C		2.057	2.061	1.901
24-h urine protein		1.296	1.305	1.242
eGFR		0.957	0.957	0.951
Oral hypoglycemic drugs use	0.299		0.424	0.343
C-statistic				
training cohort	0.626	0.986	0.986	0.986
Internal validation cohort	0.611	0.983	0.983	0.975
External validation cohort	0.506	0.863	0.860	0.878
*p* value of the Hosmer–Lemeshow test				
training cohort	0.315	0.755	0.438	0.540
Internal validation cohort	0.524	0.817	0.713	0.510
AIC				
training cohort	665.639	150.867	149.381	142.648
Internal validation cohort	167.297	44.0503	44.161	40.929

NLR: neutrophil: lymphocyte ratio; eGFR: estimated glomerular filtration rate.

### 3.4. Net reclassification improvement and integrated discrimination improvement of the models

To further evaluate the discriminatory ability of the models, we considered the following categories of the risk kidney failure within 3 years: 0% to 24.9% was low, 25.0% to 74.9% was intermediate, and 75.0% or more was high. The cutoff values used to calculate the net reclassification were 25% and 75%. The net reclassification improvement (NRI) and integrated discrimination improvement (IDI) of laboratory, lab-medication, and full models were shown in Table 7. Compared with laboratory model, lab-medication model, which added the use of oral hypoglycemic drugs, had no further improvement in either the net reclassification or integrated discrimination. The integrated discrimination improvement showed a 1.7% (95% CI, 0.7%,2.7%; *p* value, .001) improvement in full model compared with laboratory model, while the NRI of the two model was not statistically significant (*p* value, .278). The IDI between the comparison of full model and lab-medication model showed a 1.4% (95% CI, 0.6%, 2.3%; *p* value, .001) improvement, but NRI was not significant (*p* value, .057).

**Table 7.** Net reclassification improvement and integrated discrimination improvement of the models.

	Categorical NRI	*p* Value	IDI	*p* Value
Laboratory vs. lab-medication	−0.015 (–0.040–0.011)	.258	0.003 (–0.002–0.008)	.213
Full vs. laboratory	0.019 (–0.016–0.054)	.278	0.017 (0.007 –0.027)	.001
Full vs. lab-medication	0.033 (–0.001–0.068)	.057	0.014 (0.006–0.023)	.001

### 3.5. The nomogram of the optimal model

**Figure 2.**
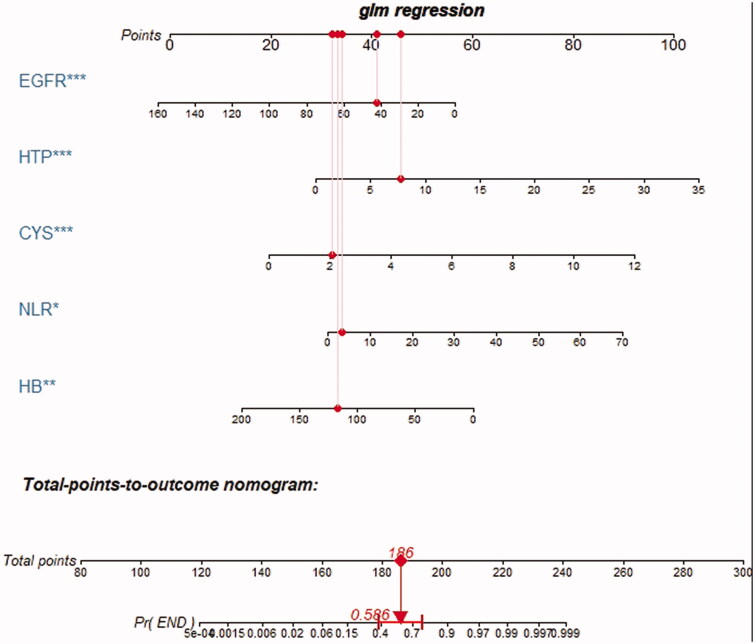
Nomogram of the laboratory model. Abbreviations: EGFR: estimated glomerular filtration rate; HTP: 24-h urine protein; CYS: serum cystatin C; NLR: neutrophil: lymphocyte ratio; HB: hemoglobin.

Based on the above analysis, laboratory model performed well in terms of both discrimination and calibration. Therefore, we regarded laboratory model, which contained hemoglobin, NLR, serum cystatin C, eGFR, 24-h urine protein as the optimal model. The nomogram to predict the probability of DKD initiating renal replacement in 3 years is shown in Figure 2. The nomogram was created based on the following five independent prognostic factors: hemoglobin level, serum cystatin C level, eGFR, 24-h urine protein level, and the NLR. A higher total number of points on the basis of the sum of the assigned number of points for each factor in the nomogram indicates a worse prognosis for the patient. For example, a patient with normal hemoglobin (120 g/L), lower eGFR (43 mL/min/1.73 m^2^), higher serum cystatin C level (2.1 mg/L), lower NLR (2.83), and very high urine protein level (7.8 g/d) would have a total of 186 points, indicating a predicted 3-year probability of the onset of renal replacement of 58.6%.

## 4. Discussion

We have developed and validated a set of risk prediction models using laboratory data that are obtained routinely in patients with DKD and could be easily integrated into a laboratory information system to predict the progression to kidney failure among patients with DKD. The laboratory model behaved good performance among the sequential models. The C-statistic for laboratory model was 0.986 in the training cohort, 0.983 in the internal validation cohort and 0.863 in the external validation cohort, showing good discriminatory performance, and the calibration was good, with *p* = .817. Our study demonstrated that lower eGFR, higher cystatin C levels, lower hemoglobin, higher NLR level and higher 24-h urine protein significantly increased the risk of renal replacement therapy in patients with DKD.

*K*-fold crossvalidation is one of the most commonly used methods of evaluating the predictive performances of a model [14]. In *K*-fold crossvalidation, (*K* − 1) fold is allocated to model development, and the residual fold is adopted for the model validation. Jung suggested the nearest integer value of log(*n*) as a rule for choosing *K* [15], while Zhang and Yang considered endorsing fivefold crossvalidation is also commonly used for model selection [14]. Consequently, we choose fivefold crossvalidation according to the sample capacity and variables available for inclusion in the model.

In recent decades, risk prediction has gained increasing attention, and several prediction models have been developed to predict CKD in the general population [5,11,16–18]. Our findings also partially overlap with those from previous studies on prognostic factors in patients with T2DM. Jardine et al. developed risk prediction models based on data from the ADVANCE cohort for a 5-year risk prediction of new-onset albuminuria and major kidney-related outcomes in patients with type 2 diabetes [16]. Chen et al. demonstrated that eGFR is a strong outcome predictor [19]. Dunkler et al. also found eGFR to be a prognostic predictor based on data from ONgoing Telmisartan Alone in combination with data from the Ramipril Global Endpoint Trial (ONTARGET) and the Outcome Reduction with Initial Glargine Intervention (ORIGIN) study [11]. In our study population, eGFR was also significant and was included in our final model. The glomerular filtration rate (GFR) is the most common prognostic factor used to predict ESRD in both clinical practice and clinical trials [20]. The CKD-EPI and MDRD equations are commonly used to estimate the GFR. One study found that the CKD-EPI gives a better estimation of the GFR compared with the MDRD [21]. Therefore, we chose the eGFR (calculated by the CKD-EPI equation) as a predictor. The Table 1 demonstrated that, there were more patients initiating renal replacement in the group of lower eGFR, which indicated that eGFR is a convincing prognostic factor for the progression of DKD to kidney failure. Simultaneously, more strong prognostic factors, the serum cystatin C levels, 24-h urine protein levels and the neutrophil: lymphocyte ratio were in detection in our prediction model, which played a vital role in our predictive model.

Proteinuria is a strong prognostic factor for the progression of DKD to kidney failure. In some studies, proteinuria was demonstrated to be a predictor of kidney failure [10,11,19]. Some studies have certified that patients with diabetes and microalbuminuria or macroalbuminuria are at a particularly high risk of death prior to reaching ESRD [22,23]. In our study population, which excluded patients who died during follow-up, the 24-h urine protein level played an important role in predicting kidney failure.

Cystatin C, a cysteine protease inhibitor, has been demonstrated to be an early renal marker in diabetic patients [24–26]. Our study suggested that the level of cystatin C is also a strong predictive factor for kidney failure. It was showed in Table 2 that the odds ratio of cystatin C was higher than serum creatine, which means that cystatin C is more sensitive in predicting the progression of diabetic kidney disease than serum creatine.

**Table 2.** Baseline laboratory parameters of the study participants.

Characteristics	Derivation cohort (*n* = 641)		External validation (*n* = 280)	*p* Value
Hemoglobin (g/L)		114 (88, 134)	105 (93, 124)	.029
NLR		2.58 (1.77, 3.88)	2.45 (1.85, 3.36)	.240
Serum creatinine (μmol/L)		101 (62, 533)	121.5 (89, 188)	.758
Serum urea (mmol/L)		8.6 (5.3, 21.4)	9.6 (6.99, 12.8)	.987
Serum uric acid (μmol/L)		330 (259, 426)	339 (287.5, 402.25)	.227
Serum cystatin C (mg/L)		1.49 (0.87, 4.04)	1.62 (1.24, 2.26)	.161
β 2 microglobulin (mg/L)		3.67 (1.68, 8.95)	3.68 (1.3, 8.29)	.472
eGFR (ml/min/1.73 m^2^)		55.17 (8.58, 106.09)	52.8 (33.95, 79.47)	.981
Serum total protein (g/L)		63.4 (57.3, 68.1)	58.65 (53.2, 65.97)	<.001
Serum albumin (g/L)		38.8 (31.9, 42.8)	32 (27.2, 38.1)	<.001
Total cholesterol (mmol/L)		4.3 (3.56, 5.12)	5.39 (4.46, 6.47)	<.001
Triglyceride (mmol/L)		1.4 (0.98, 2.06)	1.77 (1.25, 2.55)	<.001
Serum calcium (mmol/L)		2.22 (2.08, 2.32)	2.11 (1.99, 2.23)	<.001
C-reactive protein (mg/L)		2.45 (1.08, 7.1)	1.21 (0.5, 3.13)	<.001
ESR (mm/h)		21 (9, 56)	36.5 (21, 63)	<.001
24-h urine protein (g)		0.52 (0.1, 4.64)	4.35 (2.21, 7.54)	<.001
Blood glucose (mmol/L)		8.86 ± 3.25	8.87 ± 3.06	.109
Glycated hemoglobin (%)		7.4 (6.45, 9.24)	7.44 (6.3, 8.91)	.623
Bicarbonate (mmol/L)		23.1 ± 4.33	23.77 ± 3.92	.482
PH value of urine		5.96 ± 0.82	5.93 ± 0.90	.503

NLR: neutrophil: lymphocyte ratio; eGFR: estimated glomerular filtration rate; ESR: erythrocyte sedimentation rate.

In patients with DKD, the NLR was demonstrated to be associated with the 24-h urine protein level and albumin excretion in 80 Turkish patients with newly diagnosed type 2 diabetes [27]. Wheelock et al. found that the NLR predicted the loss of renal function in 941 SURDIAGENE participants during a median follow-up of 4.5 years [28]. In our study, the NLR was associated with renal replacement in patients with DKD. The odds ratio of the NLR demonstrated that it is a sensitive factor in predicting the progression of DKD.

However, a number of limitations of this study should be considered. DKD was mostly clinically diagnosed from the presence of macroalbuminuria and renal impairment in patients with diabetes. Therefore, there might be other types of kidney disease that were included. It is possible that adding pathological information may have improved the predictive ability of the models. In addition, our study population was limited to Chinese patients with type 2 diabetes and advanced CKD, so our findings may not be widely generalizable. What’s more, the time of follow-up is short. A long term prospective cohort study of multicenter is required in future.

Future studies into the prognostication of DKD should aim to optimize the inclusion criteria and stratify renal function to improve the precision of the prediction model. External validation through multicenter studies is also necessary.

## Compliance and ethical standards

### Research involving human participants

All procedures performed in studies involving human participants were in accordance with the ethical standards of the institutional and/or national research committee at which the studies were conducted, and were approved by the Clinical Research Ethics Committee of the First Affiliated Hospital of Zhengzhou University (2018-KY-82) and conform to the 1964 Helsinki declaration and its later amendments or comparable ethical standards.

### Informed consent

Participant consent was waived because of the retrospective nature of the study and the anonymous nature of clinical data.

## Supplementary Material

Supplemental Material
