# Supplementary material for: Development and validation of a predictive model for the progression of diabetic kidney disease to kidney failure
Source: Ren Fail. 2020 Jun 11;42(1):550–9. doi: 10.1080/0886022X.2020.1772294 (PMC7946054; doi:10.1080/0886022X.2020.1772294)
Supplement: Supplemental Material [file IRNF_A_1772294_SM3395.pdf]

Supplementary Table S 1 C statistics, AICs and p values for the H-L test for clinical model through 5-fold cross-validation

	**1**	**2**	**3**	**4**	**5**	**Average**
**C statistics**						
Training cohort	0.5424	0.7043	0.5447	0.6917	0.6488	0.62638
Internal validation cohort	0.5975	0.6279	0.5685	0.6251	0.6381	0.61142
**AIC**						
Training cohort	656.0846	669.4681	660.6336	673.6844	668.3239	665.63892
Internal validation cohort	175.931	164.9479	164.9241	164.0962	166.5837	167.29658
**P value of the H-L test**						
Training cohort	0.0701	0.5044	0.4457	0.2735	0.2801	0.31476
Internal validation cohort	0.3479	0.7054	0.9527	0.2606	0.3551	0.52434

Supplementary Table S 2 C statistics, AICs and p values for the H-L test for laboratory model through 5-fold cross-validation

	**1**	**2**	**3**	**4**	**5**	**Average**
**C statistics**						
Training cohort	0.9814	0.9935	0.9803	0.9887	0.9863	0.98604
Internal validation cohort	0.9842	0.9836	0.9832	0.987	0.9785	0.9833
**AIC**						
Training cohort	147.0635	160.5374	141.4085	151.8645	153.4615	150.86708
Internal validation cohort	48.91191	36.3329	50.04142	45.67093	39.29448	44.050328
**P value of the H-L test**						
Training cohort	0.584	0.9909	0.5546	0.651	0.9935	0.7548
Internal validation cohort	0.61	0.9906	0.9783	0.8909	0.6168	0.81732

Supplementary Table S 3 C statistics, AICs and p values for the H-L test for lab-medication model through 5-fold cross-validation

	**1**	**2**	**3**	**4**	**5**	**Average**
**C statistics**						
Training cohort	0.9816	0.9928	0.9788	0.9905	0.9863	0.986
Internal validation cohort	0.9833	0.9834	0.9837	0.9844	0.9779	0.98254
**AIC**						
Training cohort	147.3495	158.1786	138.6844	152.8755	149.8149	149.38058
Internal validation cohort	47.39587	38.32457	51.8203	42.0026	41.26346	44.16136

**P value of the H-L test**						
Training cohort	0.5103	0.5702	0.4368	0.1563	0.5183	0.43838
Internal validation cohort	0.2564	0.9918	0.9792	0.723	0.6136	0.7128

Supplementary Table S 4 C statistics, AICs and p values for the H-L test for full model through 5-fold cross-validation

	**1**	**2**	**3**	**4**	**5**	**Average**
**C statistics**						
Training cohort	0.9764	0.9921	0.9831	0.9917	0.9856	0.98578
Internal validation cohort	0.982	0.9749	0.9519	0.9834	0.9834	0.97512
**AIC**						
Training cohort	131.7514	152.3792	138.5493	148.0614	142.499	142.64806
Internal validation cohort	50.99661	33.43646	34.62429	43.13019	42.4558	40.92867
**P value of the H-L test**						
Training cohort	0.9816	0.2225	0.9966	0.1319	0.3671	0.53994
Internal validation cohort	0.2389	1	0.3155	0.9931	0.002302	0.5099604
